# YAP-induced MAML1 cooperates with STAT3 to drive hepatocellular carcinoma progression

**DOI:** 10.1186/s40164-025-00728-2

**Published:** 2025-12-05

**Authors:** Jiarong Li, Xi Li, Ronghao Wang, Mingyu Li, Yao Xiao

**Affiliations:** 1 Department of General Surgery, Xiangya Hospital, Central South University, Changsha, 410008 China; 2 National Clinical Research Center for Geriatric Disorders, Xiangya Hospital, Central South University, Changsha, 410008 China; 3 International Joint Research Center of Minimally Invasive Endoscopic Technology Equipment & Standards, Changsha, 410008 China; 4 Department of Geriatric Surgery, Xiangya Hospital, Central South University, Changsha, 410008 China; 5 Department of Biochemistry and Molecular Biology, School of Basic Medical Sciences, Southwest Medical University, Luzhou, 646000 China

**Keywords:** YAP, MAML1, STAT3, Hepatocellular carcinoma

## Abstract

Hepatocellular carcinoma (HCC) is a major liver malignancy and a leading cause of cancer-related mortality worldwide. Given its rising incidence and poor prognosis, there is an urgent need to elucidate the molecular mechanisms driving HCC progression and to develop novel targeted therapies. In this study, we identify MAML1 as a key contributor to HCC development. Elevated MAML1 expression strongly correlated with disease severity, whereas its knockdown suppressed HCC progression. Functionally, MAML1 promoted tumor malignancy by regulating STAT3 activity. Mechanistically, MAML1 interacted with STAT3 and enhanced its acetylation in a p300-dependent manner. Inhibition of STAT3 with a specific inhibitor attenuated MAML1-driven HCC progression. Furthermore, signaling pathway analyses revealed that YAP is the principal transcription factor regulating MAML1 expression by directly binding its promoter. Importantly, depletion of MAML1 diminished YAP-induced HCC malignancy and STAT3 activation, suggesting that YAP, MAML1, and STAT3 form a coordinated signaling axis that drives HCC progression. Collectively, these findings uncover a novel MAML1-centered signaling pathway in HCC and provide a compelling rationale for the development of MAML1-targeted clinical strategies for disease management.

**Supplementary Information:**

The online version contains supplementary material available at 10.1186/s40164-025-00728-2.

## Introduction

Hepatocellular carcinoma (HCC) ranks as the 3rd leading cause of cancer mortality worldwide and continues to threaten public health owing to its increasing incidence [1, 2]. Early-stage HCC patients are routinely treated with surgery and liver transplantation. However, an estimated 70% of cases will suffer from tumour relapse, and some diagnosed cases are identified as advanced HCC [3–5]. In this stage, immunotherapy or targeted therapy is approved to manage HCC patients and shows improved benefits [6–11]. Clinical evidence shows that the 5-year survival rate of advanced HCC patients receiving current treatments is nearly 20% [8], suggesting that the outcome is unsatisfactory. Therefore, it is necessary for clinicians or pharmacologists to develop novel targeted therapies.

STAT3, a key member of the STAT family, plays critical roles in various physiological and pathological processes, including cancer development [12–16]. JAK-mediated STAT3 phosphorylation at Y705 is considered a prerequisite for its activation [15]. Phosphorylated STAT3 subsequently translocates into the nucleus and acts as a transcription factor to regulate many genes, such as *SOCS3*, *c-M*yc and *CCND1*. In HCC, STAT3 is commonly recognized to promote HCC tumour growth, tumour metastasis, angiogenesis, antiapoptosis activity and therapy resistance [17–21]. Constitutive activation of STAT3 is also frequently observed in HCC patients and is closely associated with poor prognosis [22]. Generally, STAT3 works as a homodimer to regulate gene expression. However, in some circumstances, STAT3 can also cooperate with other transcription factors to specifically participate in certain cellular events. For example, STAT3 directly interacts with the NF-κB subunit Rel-A and promotes its p300-mediated acetylation, increasing the transcriptional activity of NF-κB [23]. Under hypoxic conditions, STAT3 binds to HIF1a to regulate VEGF expression, promoting angiogenesis [20]. Therefore, the configuration of the STAT3 complex is still a scientific topic of great interest in this area and deserves further investigation.

MAML1, the mastermind-like transcriptional coactivator 1, was originally identified as a Notch signaling coactivator [24]. Increasing evidence suggests that MAML1 can also act as a coactivator of other transcription factors. For example, MAML1 interacts with YAP/TAZ and promotes their nuclear translocation and oncogenic functions [25]. The interaction of Rel-A with MAML1 has also been reported, which potentiates NF-κB-dependent transcription [26]. Other studies have demonstrated that MAML1 is a coactivator of ERG, p300 and p53 [27–29]. These findings have helped to elucidate the roles of MAML1 in various types of diseases. Nevertheless, the role of MAML1 in HCC development and the underlying mechanism have not yet been investigated.

Our study demonstrated that MAML1, an oncogene involved in HCC development, can interact with the transcription factor STAT3 and facilitate its acetylation, allowing it to easily access the promoter regions of regulatory genes and promoting HCC progression in vitro and in vivo. In addition, MAML1 was experimentally verified as a downstream gene regulated by YAP/TEAD. Overall, the results of the present study identified the YAP-MAML1-STAT3 signaling axis in HCC and revealed that targeting this signaling pathway may alleviate HCC progression.

## Materials and methods

### Bioinformatic analyses

RNA sequencing data as well as the related clinical information were downloaded from The Cancer Genome Atlas (https://portal.gdc.cancer.gov). The “Tinyarray” R package was used to divide TCGA-LIHC samples into the “Tumor” or “Normal” group. The “pROC” R package was utilized to analyze the diagnostic value of MAML1 expression in TCGA-LIHC. The median expression level of MAML1 was used as a cutoff to divide LIHC samples into MAML1_high and MAML1_low group. For scRNA-seq analysis, the R package “Seurat” was used to analyze the HCC scRNA-seq data (GSE282701). Gene expression measurements for each cell were normalized by the total number of transcripts in the cell multiplied by a default scale factor, and the normalized values were log-transformed. For each replicate, the 2000 most highly variable genes were identified using variance stabilizing transformation (vst). All conditions were integrated using the Seurat V4 approach. To avoid obtaining results fitted too closely to a particular dataset and therefore possibly failing to fit additional data, 2000 integration anchors were first found. These anchors are then used as inputs for the dataset integration procedure. Cell clusters were visualized using t-Distributed Stochastic Neighbor Embedding (t-SNE) or Uniform Manifold Approximation and Projection (UMAP) with Seurat functions RunTSNE and RunUMAP. The VlnPlot function was used to visualize the expression of the target gene.

### Cell culture

The THLE-2, C3A, HuH7, Hep3B, HepG2, SK-Hep1, SNU-398 and HEK293T cell lines were obtained from ATCC and cultured in DMEM (Invitrogen) supplemented with 10% FBS, 1.5 g/L NaHCO_3_, 100 units/mL penicillin and 100 µg/mL streptomycin. The cells were maintained in a 5% CO_2_ humidified incubator at 37 °C. For transient transfection experiments, Lipofectamine 3000 (Invitrogen, Grand Island, NY) was used.

### Plasmid construction

The DNA sequences of interest were cloned and inserted into the PLKO or pWPI vector backbone. HA-MAML1, Flag-STAT3, YAP1 (YAP1-2γ or YAP1-504) and Flag-TEAD4 cDNA with *Pac I* and *Pme I* recognition sequence were cloned to pWPI. PLKO or pWPI with DNA sequences of interest and the plasmids psPAX2 (10 µg) and pMD2.G (10 µg) were transfected into HEK293T cells by the calcium phosphate transfection method. Forty-eight hours later, the lentivirus supernatant was harvested and filtered through a 0.45 μm filter. HCC cells were infected with the lentivirus supernatant with 8 µg/ml polybrene. Puromycin (5 µg/ml) was used to incubate shRNA-treated HCC cells for at least two weeks. Sequences of shRNAs or siRNAs were listed in Supplementary Table 1.

### MTT assay

HCC cells were seeded into 24-well plates at a density of 5000 cells and incubated with methylthiazolyldiphenyl-tetrazolium bromide (MTT) reagent at Days 0, 2, 4, and 6. Next, 500 µL of dimethyl sulfoxide (DMSO) was added, and the absorbance was measured at 570 nm. Each experimental group was assayed in triplicate to ensure reliable results.

### Transwell invasion assay

Matrigel (356235, Corning) was diluted 1:5 and seeded into the upper chambers of an 8 μm well (Corning, Inc., USA) at a volume of 100 µl per well. The plates were incubated at 37 °C until a gel formed. The cells were subsequently trypsinized and loaded into the upper chambers at a concentration of 1 × 10^5^ cells/well. DMEM supplemented with 10% FBS in the bottom chamber was used as an attractant. Twenty-four hours later, the invading cells were fixed with 75% ethanol and stained with 0.1% crystal violet. The invading cells were counted with ImageJ software.

### EdU staining

To evaluate cell proliferation, HCC cells (including MAML1-knockdown and control cells) were incubated with 10 µM EdU for 2 h to assess DNA synthesis. The cells were subsequently fixed with 4% paraformaldehyde for 15 min and permeabilized with 0.5% Triton X-100 for 10 min. The incorporated EdU was fluorescently labelled using a click reaction with azide-conjugated dyes for 30 min. Nuclei were stained with DAPI for 5 min. Fluorescence microscopy was then used to observe and quantify the percentage of EdU-positive cells.

### RNA extraction and qRT‒PCR

RNAs were isolated by the TRIzol extraction protocol. One microgram of RNA was subjected to reverse transcription using Superscript III transcriptase (Invitrogen). qRT‒PCR was conducted in a LightCycler 480 with SYBR Green to determine the mRNA expression levels of the genes of interest. GAPDH mRNA was used as an internal control. The primers used in this study are listed in Supplementary Table 1.

### Western blotting

The cells were lysed in 1X SDS buffer, and the proteins were loaded for electrophoresis on 8–12% denaturing SDS‒PAGE gels. After being transferred onto a nylon membrane, the proteins were incubated with primary antibodies, including MAML1 (PA5-18194, Invitrogen), STAT3 (4904, CST), panacetyl lysine (ab190479, Abcam), STAT3-Y705 (9131, CST), N-cadherin (4061, CST), E-cadherin (3195, CST) and GAPDH (2118, CST) antibodies, overnight at 4 °C, followed by incubation with secondary antibodies at room temperature for 1 h.

### Chromatin immunoprecipitation (ChIP)

Briefly, protein‒DNA complexes were crosslinked with 1% formaldehyde and quenched with 125 mM glycine. Then, the cells were collected in lysis buffer and subjected to sonication to obtain DNA fragments with an average length of 500 bp. After centrifugation, the supernatant was incubated with a STAT3 antibody overnight at 4 °C. Next, precleared A/G beads were used to precipitate the DNA‒protein complex, the proteins were digested with proteinase K, and the chromatin DNA was purified and subjected to RT‒qPCR assessment.

### Immunoprecipitation (IP) assay

HepG2 cells were collected and lysed in IP lysis buffer. After centrifugation, the lysates were precleared with protein A-agarose beads for 2 h, followed by incubation overnight with an anti-STAT3 antibody, anti-MAML1 or an equal amount of rabbit IgG (sc-2027, Santa Cruz) at 4 °C. The next day, the STAT3 complex was pulled down with protein A-agarose beads and subjected to western blot analysis.

### Liquid chromatography-mass spectrometry (LC-MS)

MAML1 precipitates were digested with Trypsin and Chymotrypsin enzymes. The peptides were separated by liquid chromatography (LC) and subjected to tandem mass spectrometry (MS/MS) analysis to determine their amino acid sequences. Finally, the peptide sequences were searched against a protein database by MaxQuant 2.0.1.0 software.

### Luciferase activity assay

HepG2 cells were plated in 24-well plates and transfected with pGL3-wt-MAML1 or pGL3-mut-MAML1 (100 ng/well) along with pRL-TK (1 ng/well) with or without the YAP plasmid (200 ng/well) using Lipofectamine 3000 (Invitrogen). After 24 h of transfection, the cells were lysed, and luciferase activity was assessed by a dual luciferase assay in which pRL-TK was used as the internal control. Each luciferase reading was performed in triplicate.

### In vivo studies

A total of 1 × 10^6^ SK-Hep1 cells suspended in DMEM were mixed with Matrigel (1:1, v/v) in a total volume of 200 µL and subcutaneously administered into the flank area of 6–8-week-old male nude mice. Tumour size was measured weekly by callipers. The STAT3 signaling inhibitor ruxolitinib was intraperitoneally injected every 2 days at a dose of 10 mg/kg. Finally, the mice were sacrificed for image capture and IHC staining. For the tail-vein metastasis model, a total of 1 × 10^6^ cells were suspended in PBS and injected into nude mice. Two months later, the mice were sacrificed, and tumours in the lungs were examined by HE staining. All animal experiments were conducted in accordance with the guidelines and regulations of the institutional animal care committee at our university.

### Immunohistochemical (IHC) staining

The deparaffinized and rehydrated tissue sections were treated with 3% peroxidase blocking buffer in methanol for 20 min at room temperature. Antigen retrieval was performed by boiling the sections in citrate buffer (pH 6.0) for 30 min, followed by incubation with 5% normal goat serum in PBS for 1 h. Then, the sections were blotted with an anti-Ki-67 antibody (ab15580, Abcam) or anti-MAML1 antibody (PA5-18194, Invitrogen) at 4 °C overnight. The next day, the signal was amplified by a biotin-streptavidin-HRP cascade reaction and was detected by DAB staining (SK-4100, Vectastain, Burlingame, CA).

### Statistical analysis

All the statistical analyses were conducted with GraphPad Prism 8.0 software. All values are presented as the means ± SDs, and statistical significance was determined using t tests. The chi-square test was used to analyse significant differences between the metastatic and nonmetastatic groups. *p < 0.05, **p < 0.01, ***p < 0.001 indicate significance, and “n.s.” represents nonsignificance.

## Results

### MAML1 expression is increased in HCC

**Fig. 1:**
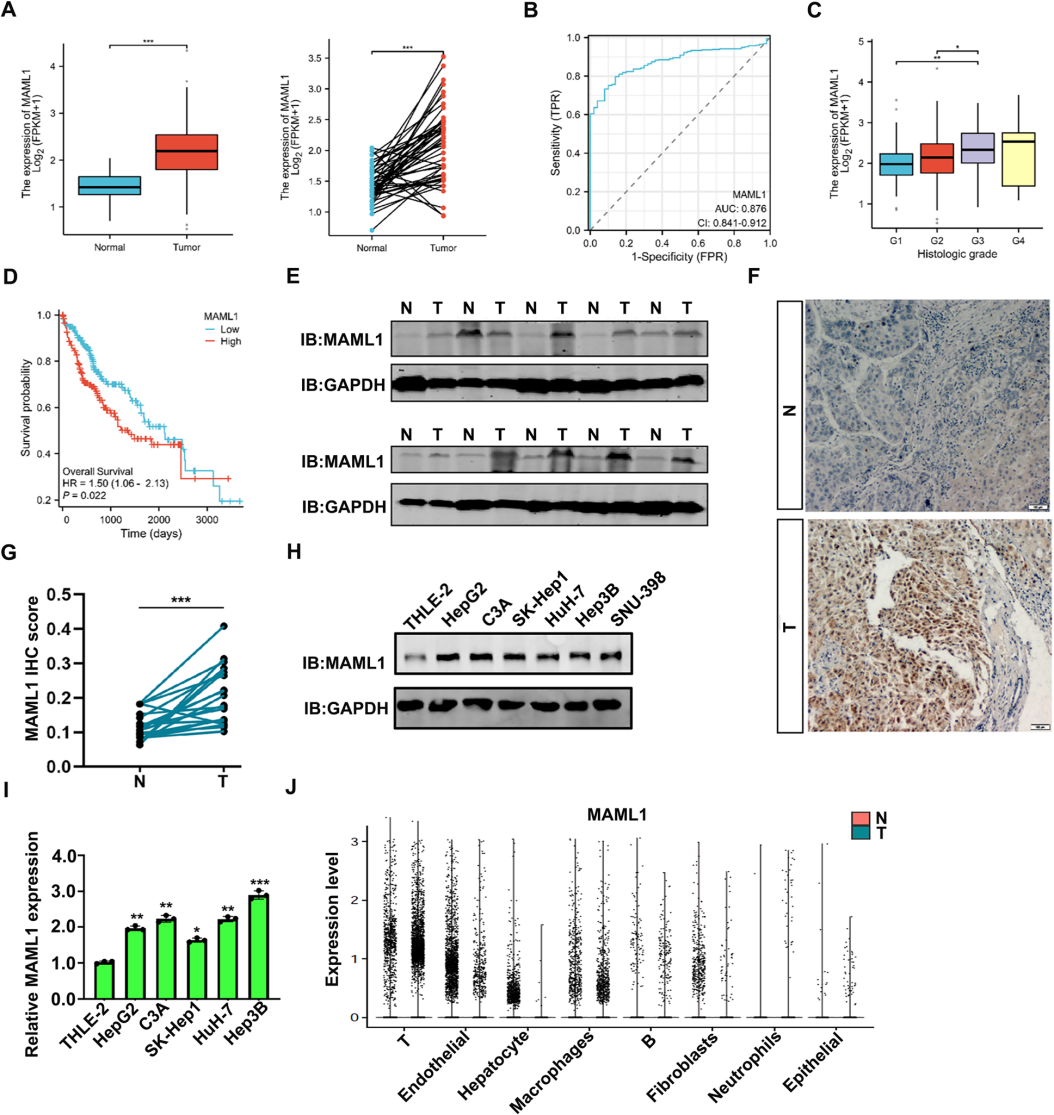
MAML1 expression is increased in HCC.** A** Compared with that in normal liver tissues, the expression level of MAML1 was increased in HCC tissues according to the TCGA-LIHC dataset. **B** ROC analysis of MAML1 in the TCGA-LIHC dataset. **C** MAML1 expression in different histologic grades from the TCGA-LIHC dataset. **D** Kaplan‒Meier survival analysis of MAML1 in the TCGA-LIHC dataset. **E** Western blot analysis of MAML1 expression in fresh HCC samples and the corresponding adjacent controls. **F-G** IHC staining analysis of MAML1 expression in HCC samples.** F**, Representative images. Scale bar = 200 μm.** G**, Statistical analysis of the MAML1 score. **H**,** I** MAML1 protein (**H**) and mRNA (**I**) levels in different HCC cell lines and the THLE-2 hepatocyte line. GAPDH was used as the internal control. **J** MAML1 expression was increased in HCC according to the available scRNA-seq dataset (singlecell.broadinstitute.org, NT-left, T-right). *p < 0.05, **p < 0.01, and ***p < 0.001

The role of the transcription cofactor MAML1 in HCC development has not yet been investigated. To this end, we first examined its expression in HCC and normal liver tissues in the TCGA-LIHC dataset. As shown in Fig. 1A, an increased level of MAML1 was clearly observed in HCC tumours compared with that in the noncancer cohorts. Notably, a higher level of MAML1 was detected in HCC patients with more than 400 ng/ml AFP, a clinical hallmark of HCC (Supplementary Fig. 1A). According to the ROC analysis, MAML1 should be considered as a diagnostic biomarker of HCC (Fig. 1B, AUC = 0.876). Further analyses revealed that MAML1 expression was further increased when HCC developed into high-grade/stage tumours (Fig. 1C & Supplementary Fig. 1B). Moreover, Kaplan‒Meier analysis revealed that HCC patients with high MAML1 expression experienced shorter overall survival (Fig. 1D, hazard ratio (HR) = 1.5, *p* = 0.022), progression-free interval (Supplementary Fig. 1C, HR = 1.67, *p <* 0.001) and disease-specific survival (Supplementary Fig. 1D, HR = 1.54, *p =* 0.057), suggesting that MAML1 is a biomarker of a poor prognosis in HCC development.

To confirm these findings, we next examined MAML1 expression in fresh HCC and adjacent liver samples and found that MAML1 protein levels were increased in 9/10 HCC samples compared with those in the adjacent controls (Fig. 1E). The IHC staining results also revealed high MAML1 expression in HCC (Fig. 1F-G). Significantly, the MAML1 was strongly correlated with HCC tumor stage (Table 1). Accordingly, MAML1 protein and mRNA levels were clearly elevated in HCC cell lines compared with those in the THLE-2 hepatocyte cell line (Fig. 1H-I). The upregulation of MAML1 in HCC was also confirmed in the available scRNA-seq dataset (GSE282701) (Fig. 1J). Together, these data suggest that MAML1 expression is increased in HCC and may contribute to HCC progression.

**Table 1:** The clinical information of HCC patients with MAML1 expression

Characteristics	Case	MAML1		*p*	χ^2^
		Low	High		
All cases	66	33	33		
Age (years)					
<60	36	16	20	0.323	0.978
≥ 60	30	17	13		
Gender					
Male	45	25	20	0.186	1.746
Female	21	8	13		
HBsAg					
Negative	12	5	7	0.523	0.407
Positive	54	28	26		
BCLC stage					
A	40	25	15	0.012	6.346
B/C	26	8	18		
Child-Pugh classification					
A	53	25	28	0.353	0.862
B	13	8	5		
AFP(g/L)					
<20	8	3	5	0.706	0.142
≥ 20	58	30	28		
Tumor size (cm)					
<5	38	23	15	0.046	3.970
≥ 5	28	10	18		
Tumor number					
Solitary	46	19	27	0.032	4.591
Multiple	20	14	6		
Liver cirrhosis					
Absence	15	9	6	0.378	0.776
Presence	51	24	27		
Tumor necrosis					
Absence	28	17	11	0.135	2.233
Presence	38	16	22		
Histological grade					
G1/G2	45	26	19	0.064	3.422
G3/G4	21	7	14		

### Influence of MAML1 on HCC malignancy

**Fig. 2:**
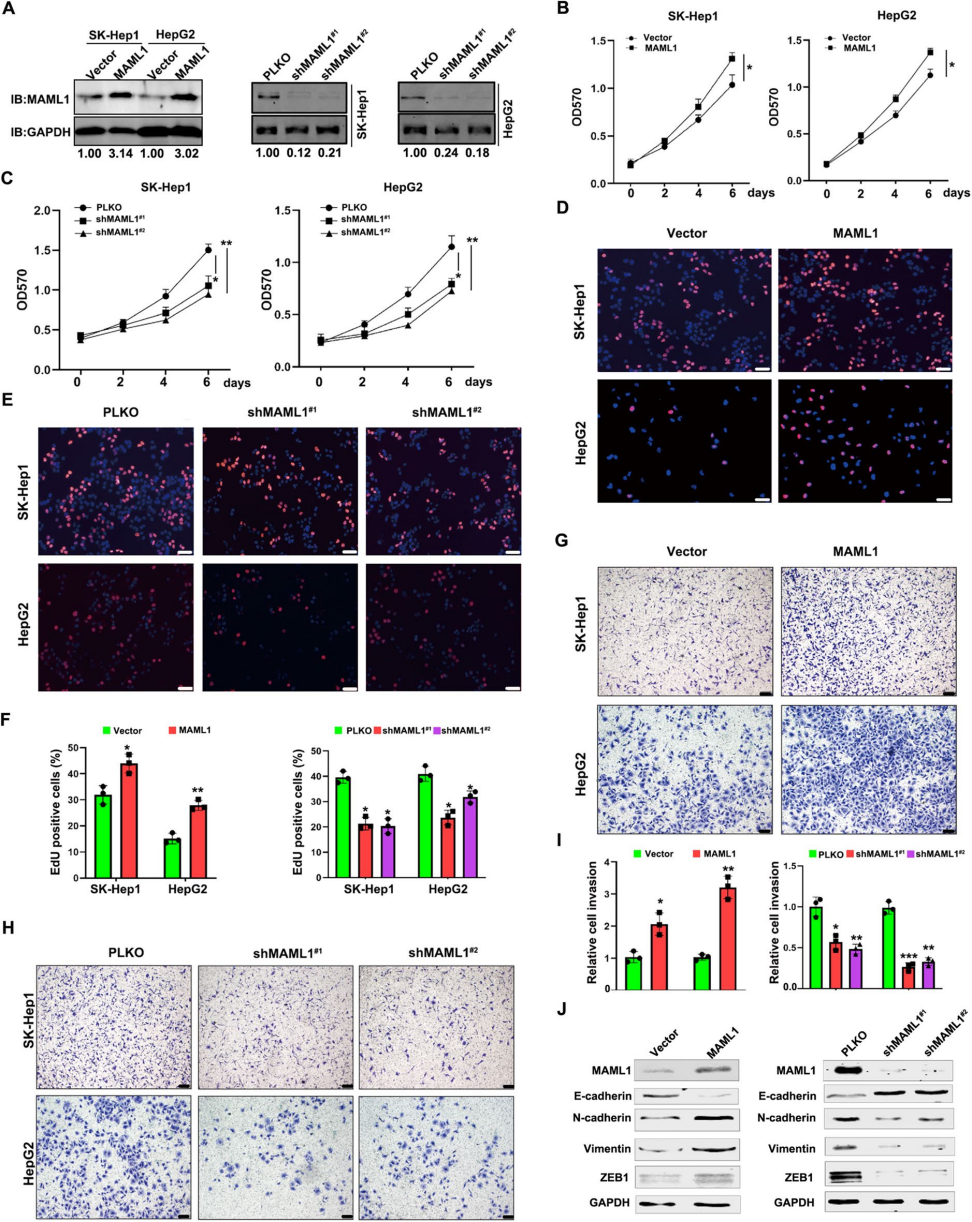
Influence of MAML1 on HCC malignancy.** A** Western blotting showing the efficiency of MAML1 expression manipulation in both HepG2 and SK-Hep1 cells. GAPDH was used as a loading control. **B** MAML1 overexpression increased the growth of HepG2 and SK-Hep1 cells. **C** MAML1 knockdown reduced the growth of HepG2 and SK-Hep1 cells. **D** EdU staining showed that MAML1 overexpression promoted the proliferation of HepG2 and SK-Hep1 cells. Scale bar: 50 μm. **E** EdU staining revealed that MAML1 knockdown decreased the proliferation of HepG2 and SK-Hep1 cells. Scale bar: 50 μm. **F** Statistical analyses of the EdU staining of** D** and** E**. **G** Chamber-Transwell invasion assays revealed that MAML1 overexpression increased the invasion of HepG2 and SK-Hep1 cells. Scale bar: 200 μm. **H** Chamber-Transwell invasion assays revealed that MAML1 knockdown decreased the invasion of HepG2 and SK-Hep1 cells. Scale bar: 200 μm. **I** Statistical analyses of the invasion assay results in** F** and** G**. **J**. Western blot analyses of E-cadherin, N-cadherin, vimentin and ZEB1 in HepG2 cells subjected to different MAML1 expression manipulations. GAPDH was used as a loading control. *p < 0.05, **p < 0.01, and ***p < 0.001

To experimentally verify the role of MAML1 in HCC development, we established stable HepG2 and SK-Hep1 cell lines with manipulated MAML1 levels, which were confirmed at both protein and mRNA levels (Fig. 2A and Supplementary 2A). The results of the MTT assay revealed that HepG2 and SK-Hep1 cells with the expression of exogenous MAML1 presented relatively faster growth rates than did the corresponding control cells (Fig. 2B). In contrast, MAML1 knockdown inhibited the growth of HepG2 and SK-Hep1 cells (Fig. 2C). EdU staining also confirmed that MAML1 expression promoted the growth of HCC cells, while its reduction inhibited HCC cell growth (Fig. 2D-F).

Additionally, the Transwell invasion assay revealed that both HepG2 and SK-Hep1 cells with MAML1 expression presented increased cell invasion activities (Fig. 2G and I). In contrast, MAML knockdown markedly suppressed the invasion of HepG2 and SK-Hep1 cells (Fig. 2H and I). Consistently, MAML1 overexpression drove epithelial‒mesenchymal transition (EMT) progression, whereas MAML1 knockdown attenuated this process (Fig. 2J), as indicated by the expression of EMT-related proteins, including N-cadherin, E-cadherin, vimentin and ZEB1, in HepG2 cells with or without manipulation of MAML1 expression. In particular, changes in MAML1 expression did not alter the apoptosis of HCC cells (Supplementary Fig. 2B-C). Collectively, these data suggest that MAML1 positively regulates HCC cell growth and invasion but has no effect on HCC cell apoptosis.

### MAML1 interacts with STAT3 and increases its acetylation

**Fig. 3:**
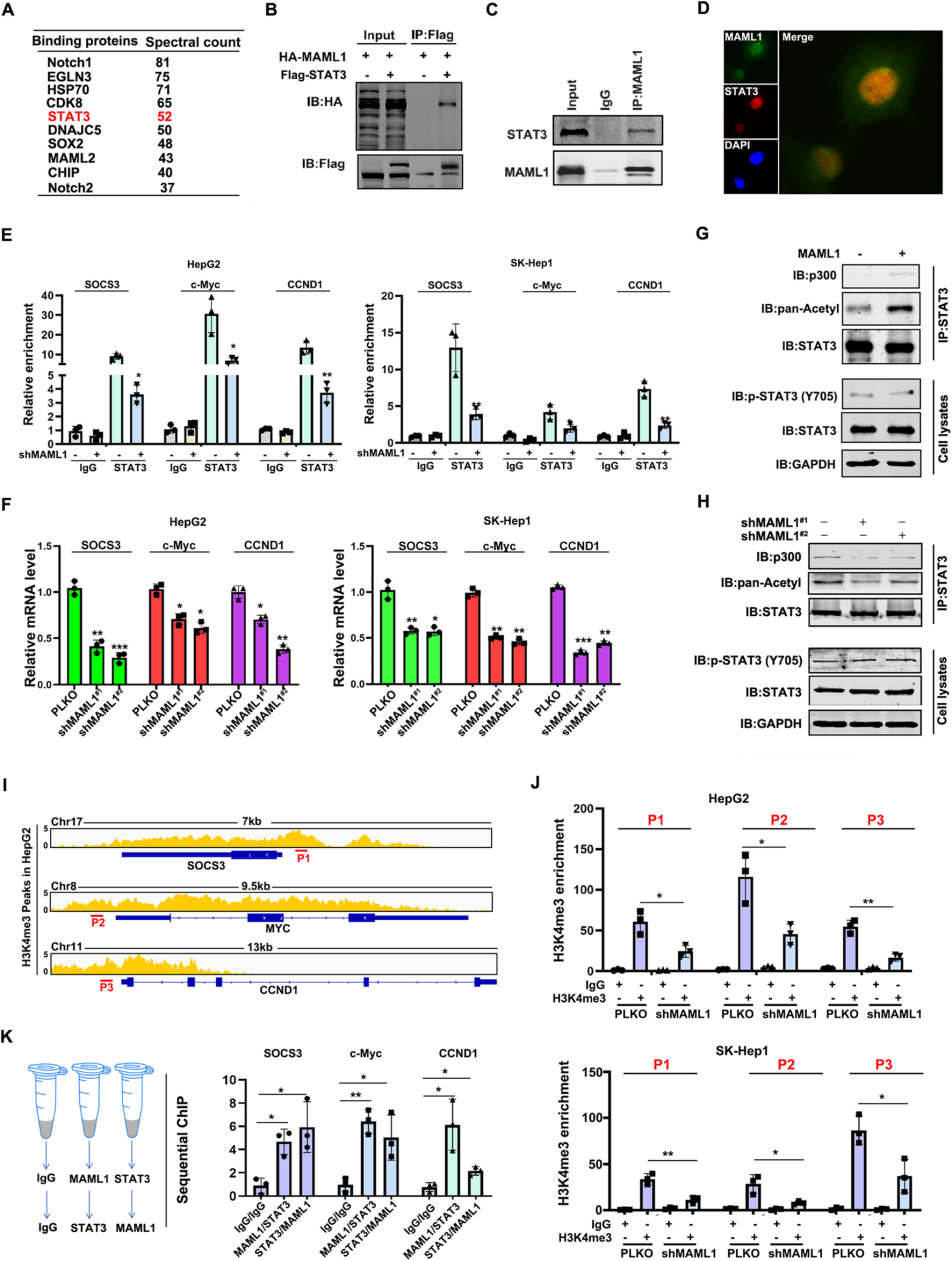
MAML1 interacts with STAT3 and increases its acetylation.** A** Top 10 MAML1-associated proteins according to mass spectrum analysis. **B-C** Immunoprecipitation assays revealed the exogenous interaction (B) and the endogenous interaction (C) between STAT3 and MAML1. **D** Immunofluorescence staining revealed the colocalization of STAT3 and MAML1 in HepG2 cells. Scale bar, 50 μm. **E** ChIP assay showing the binding capacity of STAT3 to the promoter regions of SOCS3, CCND1 and c-Myc in both HepG2 (left) and SK-Hep1 cells (right) with or without shMAML1. **F** Knockdown of MAML1 reduced the expression levels of SOCS3, CCND1 and c-Myc in both HepG2 (left) and SK-Hep1 cells (right). The expression of the detected genes was normalized to that of GAPDH mRNA. **G** An immunoprecipitation assay revealed that MAML1 recruited p300 to acetylate STAT3. **H** Immunoprecipitation revealed that MAML1 knockdown prevented p300 from acetylating STAT3. **I** H3K4me3 peaks on the promoter regions of SOCS3, CCND1 and c-Myc in HepG2 cells. **J** H3K4me3 ChIP assays demonstrated that knockdown of MAML1 reduced the promoter activity of SOCS3, CCND1 and c-Myc in HepG2 (top) and SK-Hep1 (bottom) cells. **K** Sequential ChIP revealed the STAT3/MAML1 complex in the promoter regions of SOCS3, CCND1 and c-Myc in HepG2 cells. *p < 0.05 and **p < 0.01

MAML1 is located in the nucleus of HCC cells and is thought to cooperate with other transcription factors to perform its biological function. To identify the associated transcription factors of MAML1 in HCC cells, we precipitated the MAML1 complex and subjected it to mass spectrum analysis. Among the top 10 associated proteins (Fig. 3A), STAT3 attracted our attention owing to its critical role in HCC survival and metastasis. We first confirmed the exogenous interaction of MAML1 and STAT3 in HEK293T cells (Fig. 3B) and their endogenous interaction in HepG2 cells (Fig. 3C) by an immunoprecipitation (IP) assay. Confocal imaging also revealed that MAML1 and STAT3 were colocalized in the nuclei of HepG2 cells (Fig. 3D). These data suggest that MAML1 co-locates and interacts with STAT3 in the nucleus of HCC cells, serving as a potential co-factor to regulate STAT3 activity.

These findings prompted us to investigate how MAML1 regulates STAT3 transcriptional activity. We found that the protein level, nuclear translocation, and phosphorylation status (Y705) of STAT3 were not altered upon MAML1 knockdown (Supplementary Fig. 3A-B). However, MAML1 knockdown inhibited the binding of STAT3 to the promoter regions of its downstream genes, such as SOCS3, c-Myc and CCND1 (Fig. 3E), and silenced their transcription (Fig. 3F). Mechanistically, MAML1 overexpression increased the recruitment of p300 to the STAT3 complex and facilitated STAT3 acetylation (Fig. 3G). In contrast, MAML1 knockdown inhibited the recruitment of p300 to the STAT3 complex, decreasing STAT3 acetylation (Fig. 3H). In particular, we found that the promoter activities of SOCS3, c-Myc and CCND1, presented as H3K4me3 peaks upstream of the corresponding gene locus (Fig. 3I), were apparently decreased when MAML1 was knocked down in both HepG2 and SK-Hep1 cells (Fig. 3J). By performing sequential ChIP with MAML1 and STAT3 antibodies (Fig. 3K, left), we confirmed the presence of the MAML1/STAT3 complex in the promoter regions of SOCS3, c-Myc and CCND1 (Fig. 3K, right). Notably, strong correlations between MAML1 and SOCS3, c-Myc, and CCND1 were clearly observed in the TCGA-LIHC dataset (Supplementary Fig. 3C).

Together, these findings suggest that MAML1 interacts with STAT3 and facilitates p300-mediated acetylation of STAT3, increasing its transcriptional activity.

### MAML1 drives HCC progression, which is dependent on STAT3 signaling

**Fig. 4:**
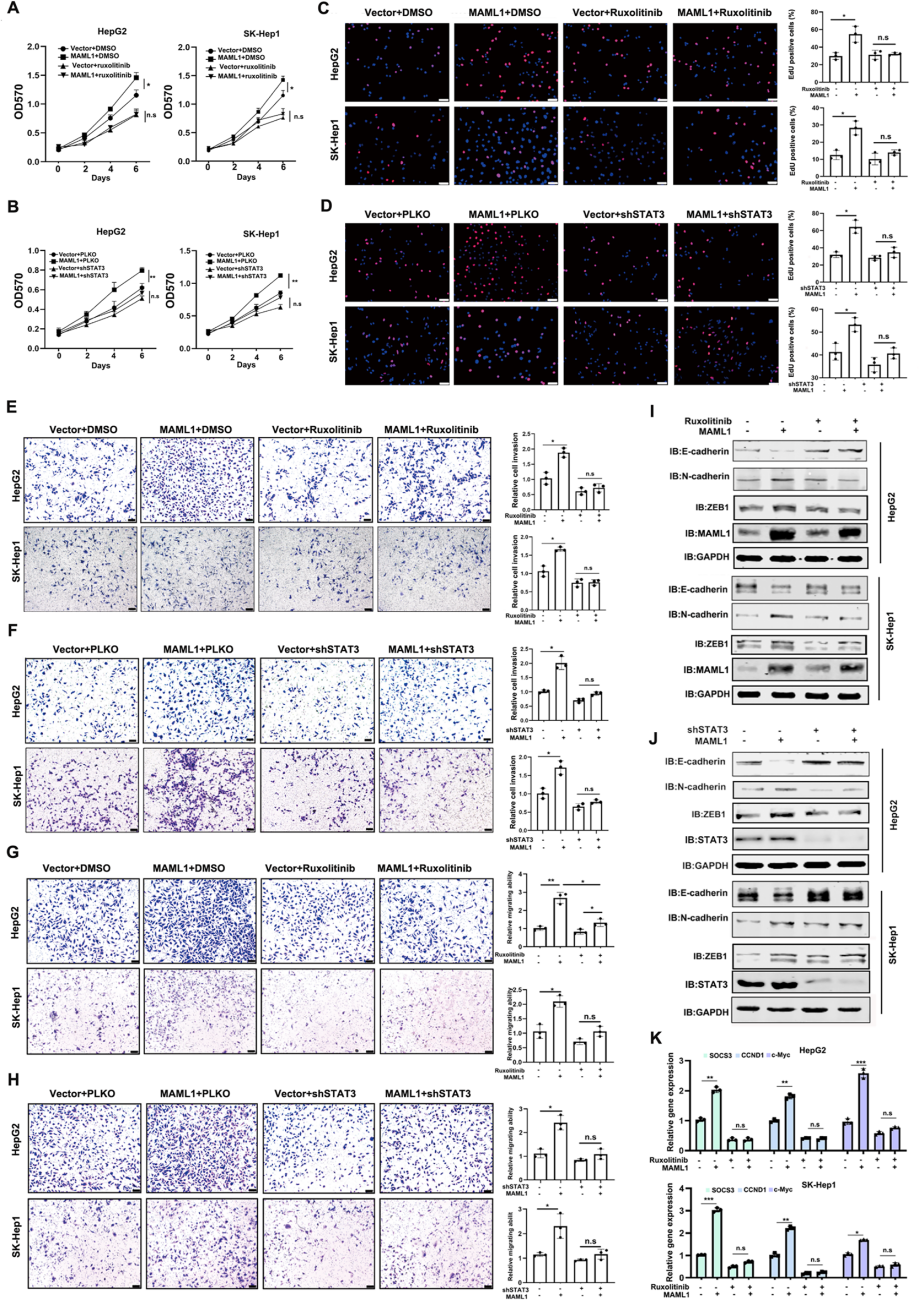
MAML1 drives HCC progression, which is dependent on STAT3 signalling. **A** MTT assays revealed that the STAT3 inhibitor ruxolitinib (1 µM) attenuated MAML1-induced growth of HepG2 (left) and SK-Hep1 (right) cells. **B** MTT assays revealed that STAT3 knockdown attenuated MAML1-induced growth of HepG2 (left) and SK-Hep1 (right) cells. **C** EdU staining assays revealed that the STAT3 inhibitor ruxolitinib (1 µM) blocked MAML1-induced proliferation of HepG2 (top) and SK-Hep1 (bottom) cells. Scale bar: 50 μm. Left, representative images of EdU-positive cells. Right, the statistical analyses of the EdU staining results. **D** EdU staining assays revealed that STAT3 knockdown blocked MAML1-induced proliferation of HepG2 (top) and SK-Hep1 (bottom) cells. Scale bar: 50 μm. Left, representative images of EdU-positive cells. Right, the statistical analyses of the EdU staining results. **E** Chamber-Transwell invasion assays revealed that the STAT3 inhibitor ruxolitinib (1 µM) attenuated MAML1-induced invasion of HepG2 and SK-Hep1 cells. Left, representative invading HCC cells. Right, the statistical analyses of the invading cells. **F** Chamber-Transwell invasion assays revealed that STAT3 knockdown attenuated MAML1-induced invasion of HepG2 and SK-Hep1 cells. Left, representative invading HCC cells. Right, the statistical analyses of the invading cells. **G** Chamber-Transwell migration assays revealed that the STAT3 inhibitor ruxolitinib (1 µM) attenuated MAML1-induced cell migration in HepG2 and SK-Hep1 cells. Left, representative migrating HCC cells. Right, the statistical analyses of the migrating cells. **H** Chamber-Transwell migration assays revealed that STAT3 knockdown attenuated MAML1-induced cell migration in HepG2 and SK-Hep1 cells. Left, representative migrating HCC cells. Right, the statistical analyses of the migrating cells. **I** Western blot analyses indicated that the STAT3 inhibitor ruxolitinib (1 µM) blocked MAML1-induced EMT progression in HepG2 (top) and SK-Hep1 (bottom) cells. GAPDH was used as an internal control. **J** Western blot analyses indicated that STAT3 knockdown blocked MAML1-induced EMT progression in HepG2 (top) and SK-Hep1 (bottom) cells. GAPDH was used as an internal control. **K** RT‒qPCR assays revealed that the STAT3 inhibitor ruxolitinib (1 µM) attenuated MAML1-induced increases in the expression levels of SOCS3, CCND1 and c-Myc in HepG2 (top) and SK-Hep1 (bottom) cells. *p < 0.05, **p < 0.01, and ***p < 0.001, and “n.s.” indicates no significance

However, whether the biological functions of MAML1 in HCC development rely on STAT3 signaling was unknown. To this end, we utilized the STAT3 inhibitor ruxolitinib to treat MAML1-overexpressing HepG2 and SK-Hep1 cells. As shown in Fig. 4A, MAML1-induced growth of HepG2 and SK-Hep1 cells was markedly impaired by ruxolitinib treatment. Moreover, manipulation of STAT3 expression by shRNA also blocked MAML1-induced growth of HepG2 and SK-Hep1 cells (Fig. 4B). EdU staining also revealed that MAML1-induced proliferation of HepG2 and SK-Hep1 cells was blocked by either ruxolitinib or shSTAT3 (Fig. 4C-D). Accordingly, MAML1-induced invasion and migration of HepG2 and SK-Hep1 cells were attenuated in the presence of ruxolitinib or shSTAT3 (Fig. 4E-H). Moreover, ruxolitinib exposure or STAT3 knockdown apparently inhibited MAML1-induced EMT progression, as determined by western blot analysis of E-cadherin, N-cadherin and ZEB1 expression levels (Fig. 4I-J). In the presence of ruxolitinib, MAML1 failed to increase the expression levels of STAT3-regulated CCND1, SOCS3 and c-Myc (Fig. 4K).

We also confirmed that ruxolitinib treatment enabled to reduce the phosphorylation and acetylation levels of STAT3, implying STAT3 phosphorylation is a prerequisite for STAT3 acetylation in certain circumstance (Supplementary Fig. 4A). To confirm the specific role of MAML1 mediated STAT3 acetylation, we introduced STAT3 K685R (non-acetylatable mutant) or STAT3 K685Q (acetylatable mutant) into MAML1 depleted HCC cells, to examine whether it can recover MAML1 deficiency mediated phenotypes. As shown in Supplementary Fig. 4B-C, STAT3 K685Q but not K685R enabled to recover the reduced cell invasion and cell proliferation caused by MAML1 knockdown, highly suggesting that it is the STAT3 acetylation conveying the biological functions of MAML1 to HCC cells. Moreover, the ChIP assay revealed that STAT3 K685Q had much stronger ability to occupy the promoter regions of SOCS3, CCND1 and c-Myc (Supplementary Fig. 4D), implying MAML1 mediated STAT3 acetylation enhances its DNA binding ability.

Together, these data suggest that the oncogenic role of MAML1 in HCC at least partially relies on STAT3 acetylation.

### YAP regulates MAML1 expression in HCC

**Fig. 5:**
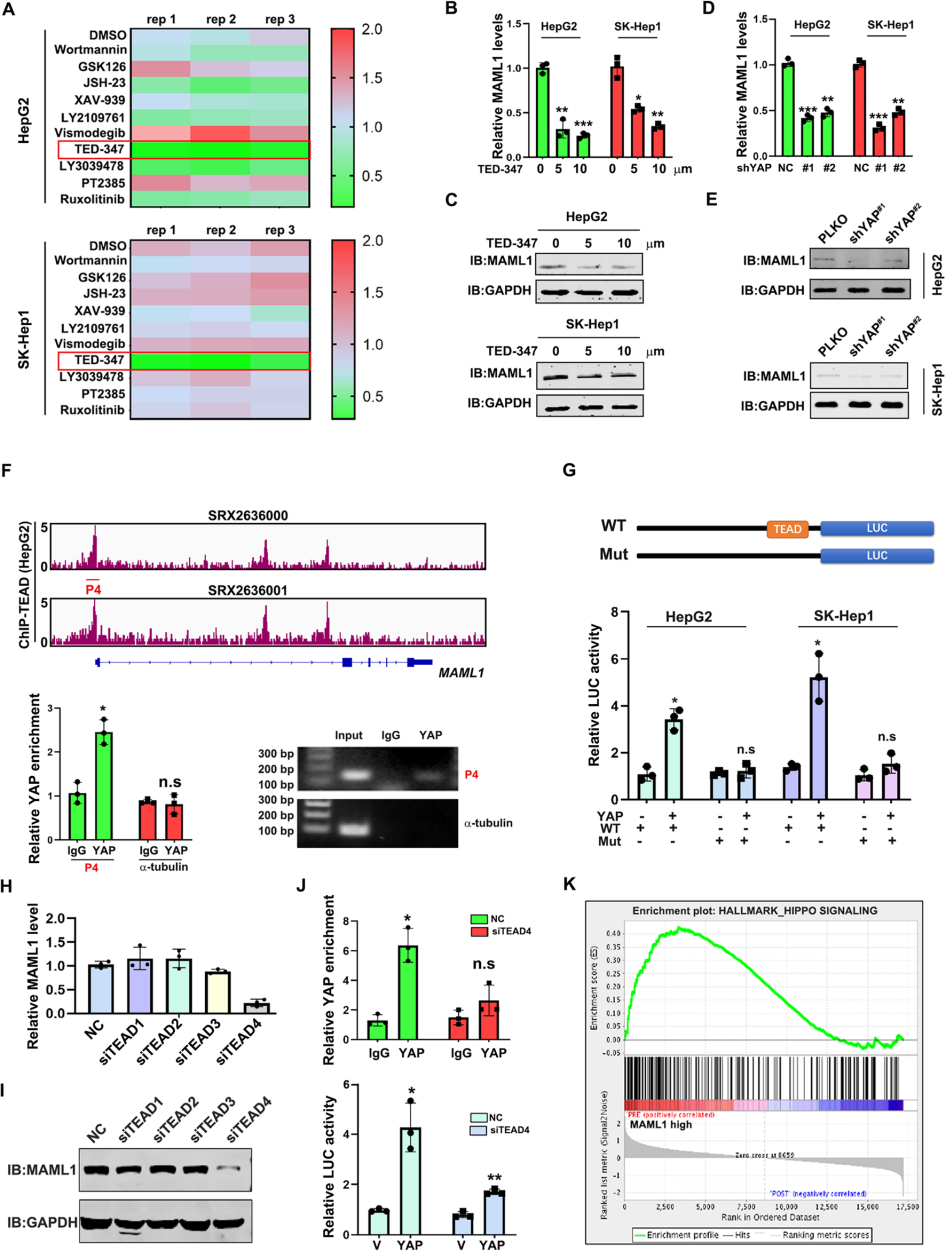
YAP regulates MAML1 expression in HCC.** A** Heatmap derived from RT‒qPCR assays of MAML1 expression in HepG2 (top) and SK-Hep1 (bottom) cells treated with different pathway inhibitors. GAPDH mRNA was used to normalize the expression of genes of interest. **B-C** The YAP inhibitor TED-347 suppressed MAML1 at both the mRNA (**B**) and protein (**C**) levels in HCC cells. GAPDH was used as an internal control. **D-E** YAP knockdown by siRNAs suppressed MAML1 at both the mRNA (**D**) and protein (**E**) levels in HCC cells. GAPDH was used as an internal control. **F** YAP directly regulated MAML1 expression by binding to its promoter region. Top, schematic cartoon showing the TEAD binding site in the promoter region of MAML1. Bottom, ChIP assays in HepG2 cells revealed that YAP directly bound to the promoter of MAML1. MAML1 on the promoter region of a-tubulin was used as a negative control. **G** Luciferase assays revealed that YAP increased the promoter activity of MAML1. **H** RT‒qPCR assay showing the effect of each TEAD member on MAML1 expression. **I** Western blotting was used to determine the effect of each TEAD family member on MAML1 expression. GAPDH was used as a loading control. **J** Top, a ChIP assay revealed that TEAD4 knockdown prevented the enrichment of YAP in the promoter region of MAML1. Bottom, the results of the luciferase assay revealed that TEAD4 blocked YAP-induced MAML1 transcription. **K** GSEA revealed a correlation between MAML1 expression and Hippo signaling in the TCGA-LIHC dataset. *p < 0.05, **p < 0.01, and ***p < 0.001, and “n.s.” indicates no significance

To identify the signaling pathway responsible for MAML1 expression in HCC, we treated HepG2 and SK-Hep1 cells with various inhibitors and assessed MAML1 expression by RT‒qPCR. As shown in Fig. 5A-C, the YAP inhibitor TED-347 consistently suppressed MAML1 expression in HepG2 and SK-Hep1 cells at both the protein and mRNA levels. Similarly, YAP shRNAs (Supplementary Fig. 5A) decreased MAML1 protein and mRNA levels (Fig. 5D-E). By examining the available ChIP-seq dataset (https://chip-atlas.org/peak), we found that there is a TEAD binding site in the promoter region of MAML1 in HepG2 cells (Fig. 5F), suggesting that YAP may directly regulate MAML1 expression by binding to the TEAD protein. To test this hypothesis, we precipitated the YAP–DNA complex and performed RT‒qPCR. The data revealed that YAP was enriched in the promoter region of MAML1 (Fig. 5F). To test whether this binding is sufficient to regulate the promoter activity of MAML1, we constructed a pGL-3 luciferase vector containing either the wild-type or TEAD-deleted MAML1 promoter region and performed a luciferase activity assay. As shown in Fig. 5G, YAP increased the luciferase activity of the wild-type construct but had little effect on the TEAD deletion control. To identify which TEAD member is required for YAP-induced MAML1 expression, we individually knocked them down with specific siRNAs (Supplementary Fig. 5B) and examined MAML1 expression, which revealed that TEAD4 knockdown decreased MAML1 expression at both the mRNA and protein levels (Fig. 5H-I). In contrast, TEAD4 overexpression was sufficient to drive MAML1 expression in HepG2 cells (Supplementary Fig. 5C). Consistently, TEAD4 deficiency blocked YAP recruitment to the MAML1 promoter and attenuated YAP-induced MAML1 transcription (Fig. 5J). Notably, GSEA revealed that MAML1 expression was highly correlated with Hippo signaling (Fig. 5J) and that YAP expression was strongly correlated with MAML1, SOCS3, CCND1 and c-Myc expression according to the TCGA-LIHC dataset (Supplementary Fig. 5D). Collectively, these data suggest that YAP physically binds to the promoter region of MAML1 to increase its transcription.

### YAP relies on MAML1 to facilitate HCC development

**Fig. 6:**
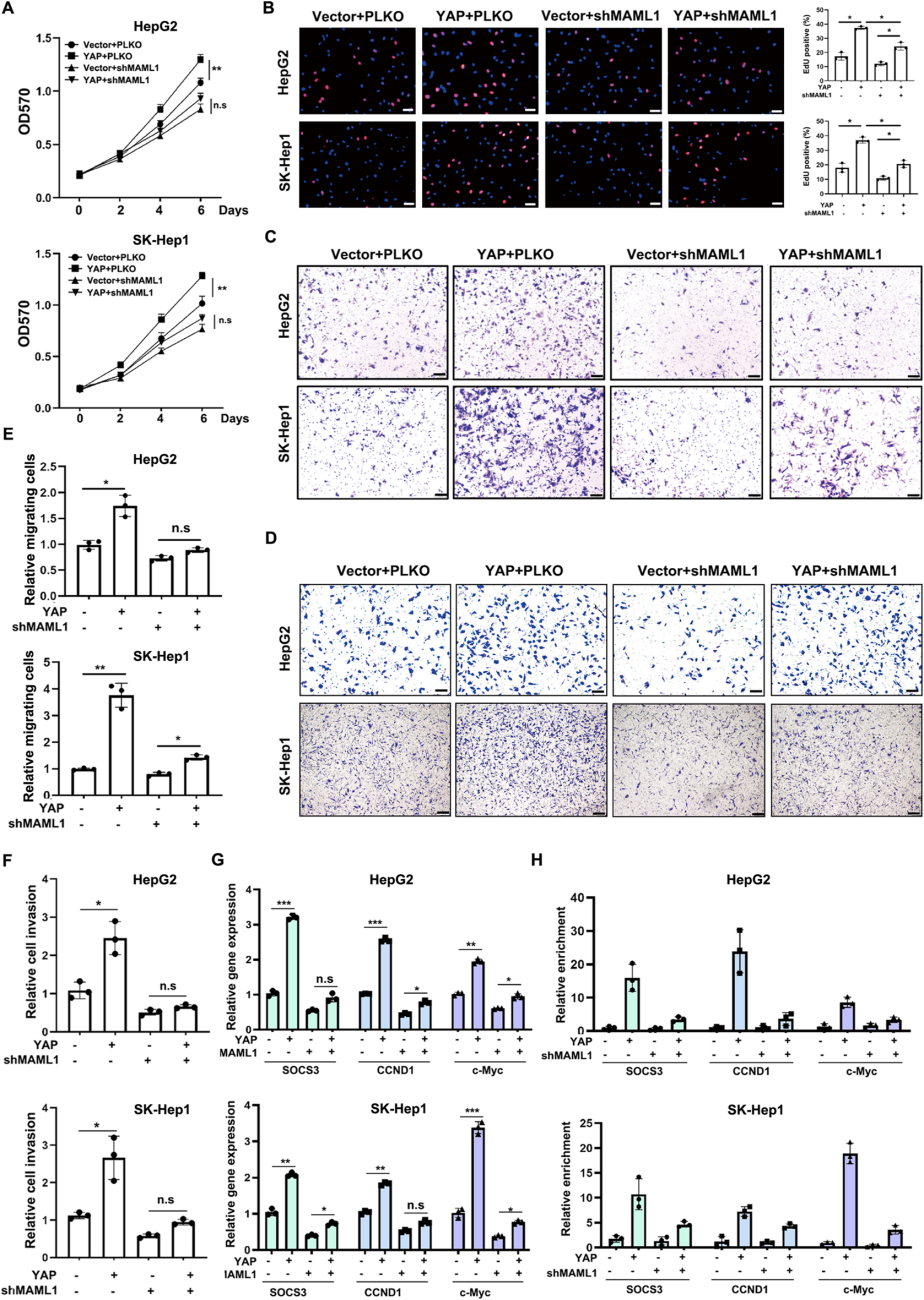
YAP relies on MAML1 to facilitate HCC development.** A** MTT assays revealed that MAML1 knockdown attenuated the YAP-induced growth of HepG2 (left) and SK-Hep1 (right) cells. **B** EdU staining assays revealed that MAML1 knockdown blocked the YAP-induced growth of HepG2 (top) and SK-Hep1 (bottom) cells. Scale bar: 50 μm. Left, representative images of the EdU-positive cells. Right, the statistical analyses of the EdU staining results. **C** Chamber-Transwell migration assays revealed that MAML1 knockdown attenuated the YAP-induced migration of HepG2 and SK-Hep1 cells. **D** Chamber-Transwell invasion assays revealed that MAML1 knockdown attenuated the YAP-induced invasion of HepG2 and SK-Hep1 cells. **E** Statistical analyses of the migrating cells in** C**. **F** Statistical analyses of the invading cells in** D**. **G** RT‒qPCR assays revealed that MAML1 knockdown attenuated the YAP-induced expression of SOCS3, CCND1 and c-Myc in HepG2 (top) and SK-Hep1 (bottom) cells. **H** ChIP assays revealed that MAML1 knockdown attenuated YAP-induced STAT3 enrichment at the promoter regions of SOCS3, CCND1 and c-Myc in HepG2 (top) and SK-Hep1 (bottom) cells. *p < 0.05, **p < 0.01, and ***p < 0.001, and “n.s.” indicates no significance

YAP has been shown to promote HCC development. However, whether the contribution of YAP to HCC progression is MAML1 dependent has not been investigated. To this end, we ectopically expressed YAP in HepG2 and SK-Hep1 cells and then decreased MAML1 expression by shRNA. MTT and EdU staining assays demonstrated that the YAP-induced growth and proliferation of HepG2 and SK-Hep1 cells were clearly inhibited by MAML1 knockdown (Fig. 6A-B). In addition, the YAP-induced invasion and migration of HepG2 and SK-Hep1 cells were attenuated by MAML1 knockdown (Fig. 6C-F). Interestingly, the YAP-induced expression of SOCS3, CCND1 and c-Myc was markedly suppressed by MAML1 knockdown (Fig. 6G), which was accompanied by decreased STAT3 enrichment in the promoter regions of SOCS3, CCND1 and c-Myc (Fig. 6H). We also confirmed that YAP overexpression could increase the STAT3 acetylation levels but not its phosphorylation levels in HepG2 cells, which was diminished when MAML1 was depleted by shRNA, strengthening the existence of YAP-MAML1-STAT3 axis in HCC cells (Supplementary Fig. 5E). Overall, these data suggest that YAP at least partially relies on MAML1-STAT3 signaling to facilitate HCC development.

### Targeting MAML1 suppresses HCC growth and metastasis

**Fig. 7:**
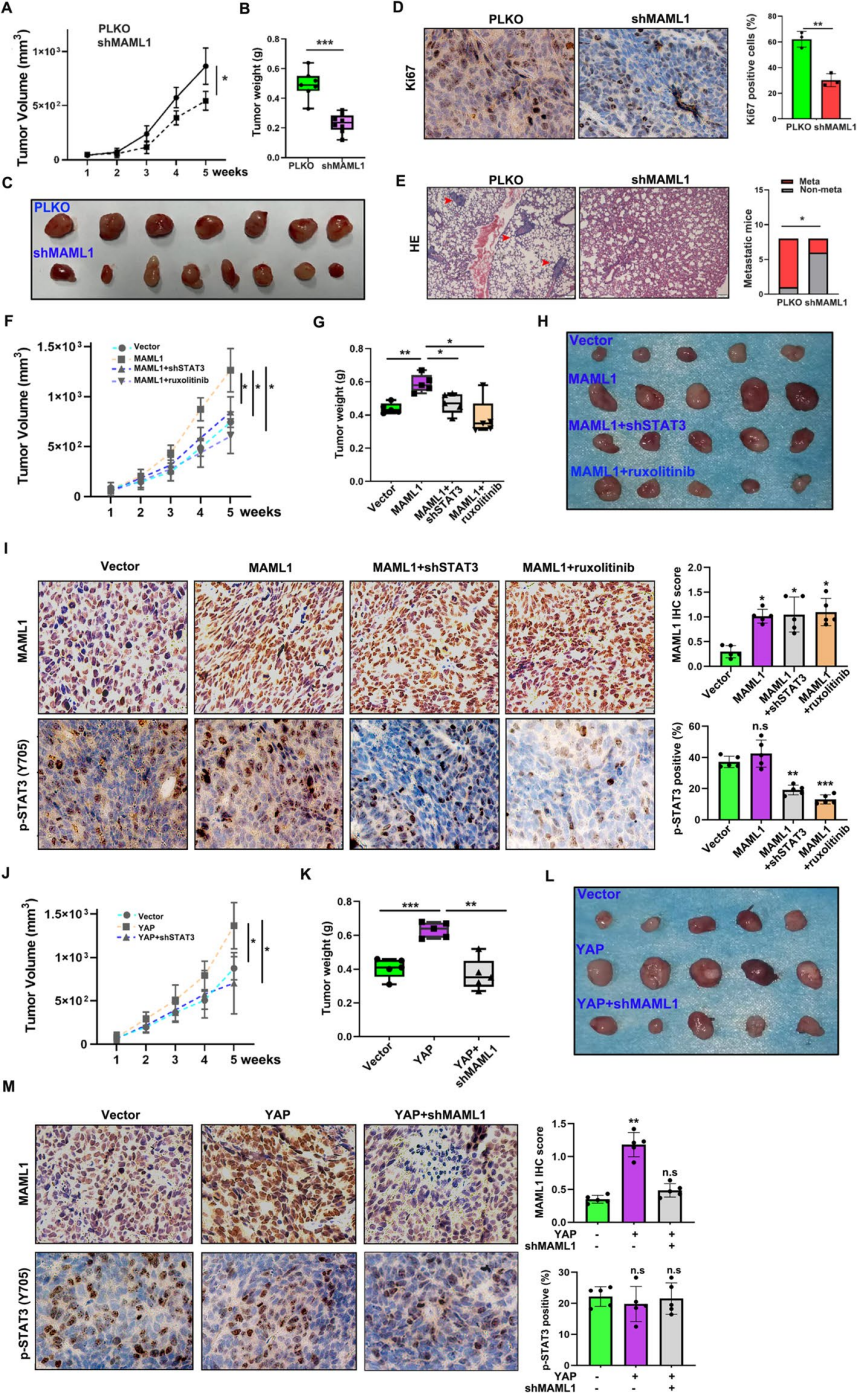
Targeting MAML1 suppresses HCC growth and metastasis.** A** Growth curves of SK-Hep1 PLKO and shMAML1 HCC tumours. **B** Tumour weights of SK-Hep1 PLKO and shMAML1 HCC tumours after 5 weeks. **C** PLKO and shMAML1 tumour images at the endpoint. **D** IHC staining for Ki67. Scale bar: 200 μm. Left, representative images of the Ki67 staining. Right, quantification of the Ki67 staining. **E** A tail-vein metastasis model suggested that MAML1 inhibition suppressed HCC metastasis. Left, representative images of the metastatic foci; Right, quantification of the metastatic mice. Scale bar: 200 μm. **F-H** Growth curves (**F**), tumour weights (**G**) and tumour images (**H**) of SK-Hep1 tumours subjected to various treatments: vector; MAML1; MAML1 + shSTAT3; and MAML1 + ruxolitinib. **I** IHC staining assays for MAML1 and p-STAT3 (Y705) in tumours from H. Left, representative images. Right, quantification of the IHC staining. **J-L** Growth curves (**J**), tumour weights (**K**) and tumour images (**L**) of SK-Hep1 tumours subjected to various treatments: vector; YAP; and YAP + shMAML1. **M** IHC staining assays for MAML1 and p-STAT3 (Y705) in tumours from L.The significance levels are denoted as *p < 0.05, **p < 0.01 and ***p < 0.001

To test whether MAML1 inhibition has the ability to suppress HCC tumour growth, we inoculated PLKO and shMAMLl1 SK-Hep1 cells into the flank area of 6-week-old male mice and monitored tumour growth weekly. We noticed that shMAML1 SK-Hep1 tumours grew at a slower rate compared to those in the PLKO cohorts (Fig. 7A). The endpoint tumour sizes of the shMAML1 HCC tumours were clearly smaller than those of the PLKO control tumours (Fig. 7B-C). IHC staining assays confirmed MAML1 was successfully reduced in shMAML1 SK-Hep1 tumours as compared to the PLKO counterparts (Supplementary Fig. 5F). Ki67 staining also verified that MAML1-depleted HCC tumours had fewer positive cells, implying an inhibition of cancer proliferation (Fig. 7D). We also constructed a tail-vein metastasis model to examine whether targeting MAML1 could suppress HCC tumour metastasis. The results revealed that HCC tumours with MAML1 knockdown had a weaker capacity to colonize the lungs than control tumours did (Fig. 7E). These data suggest that targeting MAML1 can suppress HCC growth and metastasis. Moreover, we confirmed that MAML1 could drive HCC tumour growth in vivo, which could be blocked by the inhibition of STAT3 signaling either by ruxolitinib administration or shRNA (Fig. 7F-I), suggesting that the tumour-promoting role of MAML1 is dependent on STAT3 signaling. Finally, we confirmed in vivo that the oncogenic role of YAP in HCC relied on its regulation of MAML1 (Fig. 7J-L). Significantly, YAP could promote MAML1 expression without altering STAT3 phosphorylation (Fig. 7M), implying YAP induced MAML1 expression specifically enhances STAT3 acetylation to drive HCC development, which was consistent with our in vitro findings.

**Fig. 8:**
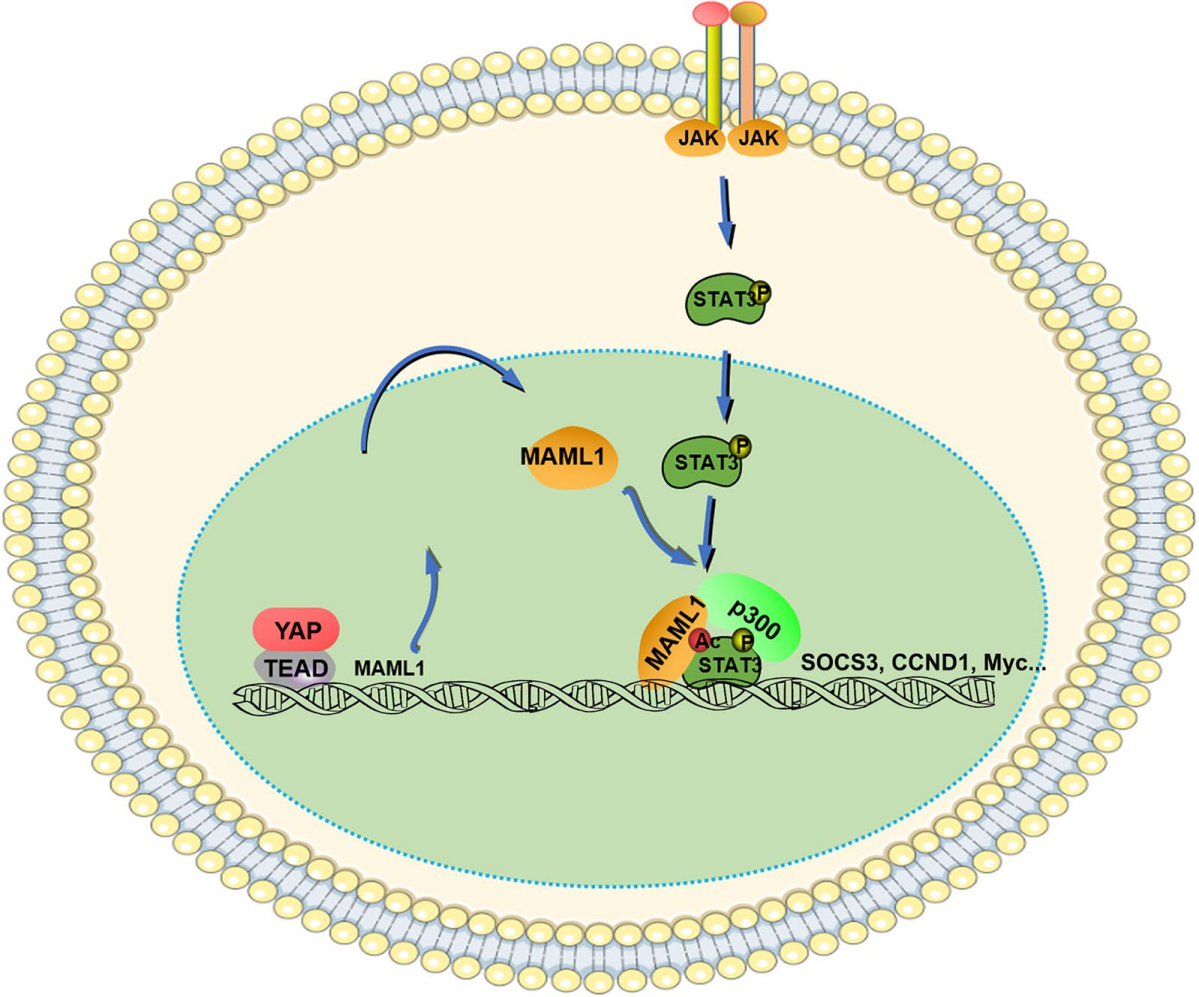
Schematic depiction of how YAP-induced MAML1 cooperates with STAT3 to promote HCC growth and metastasis

Overall, our study demonstrated that YAP-induced MAML1 promotes HCC progression by interacting with STAT3 and activating STAT3 signaling. MAML1 recruits p300 to the STAT3 complex and facilitates its acetylation (Fig. 8). Targeting the YAP-MAML1-STAT3 signaling axis is an ideal therapeutic strategy to overcome HCC progression.

## Discussion

Numerous signaling molecules have been identified as key players that drive HCC development [30–35]. Nevertheless, the exact underlying mechanisms responsible for HCC progression remain to be explored. A previous study demonstrated that MAML1 is a poor prognostic biomarker of HCC. However, the exact role of MAML1 during HCC tumorigenesis has not yet been investigated. In this study, MAML1 was found to be increased in HCC tissues, and its expression was strongly correlated with HCC prognosis. The in vitro and in vivo evidence suggested that MAML1 functions as a STAT3 coregulator to drive HCC progression. Mechanistically, MAML1 interacted with STAT3 to increase its transcriptional activity via p300-mediated acetylation. Consequently, acetylated STAT3 had a greater affinity for binding to DNA elements, increasing the transcription of downstream genes. STAT3 inhibition indeed dampened the oncogenic effects of MAML1 on HCC cells. We also found that the induction of MAML1 in HCC was due to the regulation of YAP. Overall, this study identifies MAML1 as an oncogenic factor controlling HCC development, providing an alternative therapeutic strategy for HCC prevention.

MAML1, a transcriptional coregulator, has been shown to participate in the development of various diseases [25, 26, 29]. Nevertheless, the exact role of MAML1 in HCC has not yet been investigated. In this study, our group is the first to demonstrate that MAML1 is also causally related to HCC progression. Our results revealed that MAML1 acts as a coregulator of STAT3 and increases its transcriptional activity, promoting HCC development. However, we observed that STAT3 inhibition did not completely block MAML1-induced growth and invasion of HCC cells, suggesting that MAML1 has other biological functions independent of STAT3. Therefore, further investigations of MAML1-associated signaling pathways in HCC are needed.

The aetiology of HCC is a multifactor controlled process [36]. Among these factors, hepatitis B virus (HBV) and hepatitis C virus (HCV) are always considered the primary causes [37]. In this context, HCC is also viewed as an inflammation-related disease. IL-6, an inflammatory cytokine, is highly expressed in HCC patients and is implicated in HCC occurrence and development via the regulation of IL-6R/gp130/STAT3 signaling [17, 38]. Moreover, constitutive activation of STAT3 is observed in approximately 60% of HCC patients compared with healthy controls, possibly owing to elevated IL-6 levels or dysfunction of STAT3 negative regulators [39]. Upregulation of coregulators is another means to increase the activity of transcription factors. Given that MAML1 is a coregulator of STAT3, we believe that the induction of MAML1 also partially accounts for the constitutive activation of STAT3 in HCC and that targeting MAML1 with its inhibitor may overcome HCC progression by reducing STAT3 transcriptional activity. This study also demonstrated that MAML1 facilitates STAT3 acetylation, which increases its ability to bind DNA. Intriguingly, MAML1 had no effect on STAT3 phosphorylation, suggesting that STAT3 phosphorylation is a pioneer process ahead of its acetylation. However, whether STAT3 phosphorylation is required for MAML1 binding remains to be explored.

Our findings revealed the upregulation of MAML1 in HCC. The focused suppression of signaling pathways by specific inhibitors revealed that YAP was the main biofactor responsible for MAML1 induction in HCC. YAP directly bound to the promoter region of MAML1 and drove its transcription. As the downstream executor of Hippo signaling, YAP plays critical roles in HCC tumorigenesis and therapeutic resistance [40–43]. For example, genetic activation of YAP by liver-specific deletion of Sav1, an upstream negative regulator of YAP, promotes HCC development. Additionally, YAP can cooperate with ATF4 to drive sorafenib resistance in HCC [44–47], thus leading to the combination of the YAP inhibitor CA3 with sorafenib to treat HCC cells. Given the importance of YAP in HCC development, targeting YAP has become an ideal therapeutic strategy to fight HCC. As a YAP/TEAD interaction inhibitor, IAG933 is now being tested in clinical trials for solid tumours [48]. The determination of the clinical outcome of YAP inhibitors in HCC patients will be fascinating. Our study revealed that the oncogenic effects of YAP on HCC at least partially rely on its regulation of MAML1, suggesting that therapeutics targeting MAML1 may also attenuate the biological function of YAP.

In summary, MAML1, which is induced by YAP in HCC, cooperates with STAT3 to promote HCC survival and metastasis. Thus, MAML1 inhibition is considered a promising strategy to prevent HCC progression.

## Supplementary Information

Below is the link to the electronic supplementary material.

Supplementary Material 1

## Data Availability

All raw data and raw materials will be provided if there is a reasonable request.
