# Supplementary material for: YAP-induced MAML1 cooperates with STAT3 to drive hepatocellular carcinoma progression
Source: Exp Hematol Oncol. 2025 Dec 5;14:137. doi: 10.1186/s40164-025-00728-2 (PMC12696947; doi:10.1186/s40164-025-00728-2)
Supplement: Supplementary file 1 — Supplementary Material 1 [file 40164_2025_728_MOESM1_ESM.pdf]

Supplementary Data& Legends

Supplementary Figure 1

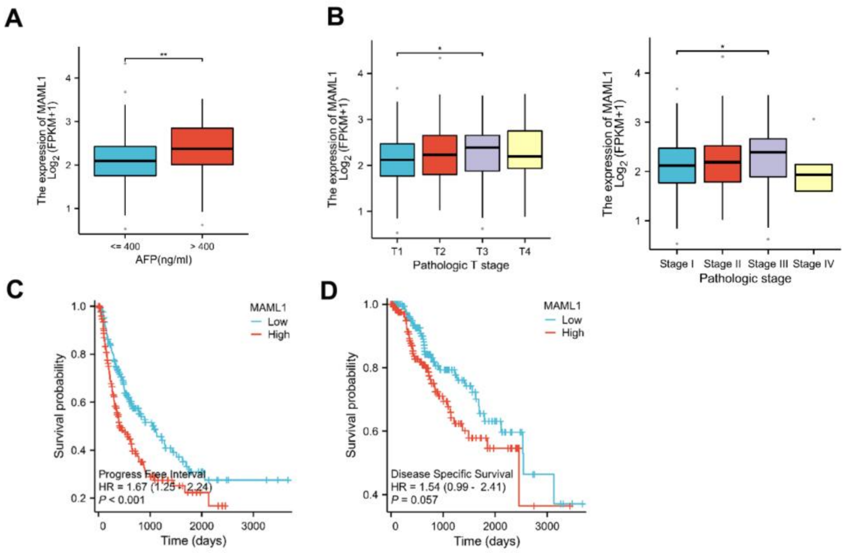

Supplementary Fig. 1 MAML1 expression level was correlated with AFP levels, tumor stage, and patient survival in HCC. A. MAML1 expression levels in TCGA-LIHC patients with different concentrations of AFP. **B.** MAML1 expression levels in different pathological stages of TCGA-LIHC patients. **C-D.** HCC patients with high MAML1 expression had shorter progression-free intervals (C) and disease-specific survival (D) than did HCC patients with low MAML1 expression. Significance levels are denoted as *p < 0.05 and **p < 0.01.

Supplementary Figure 2

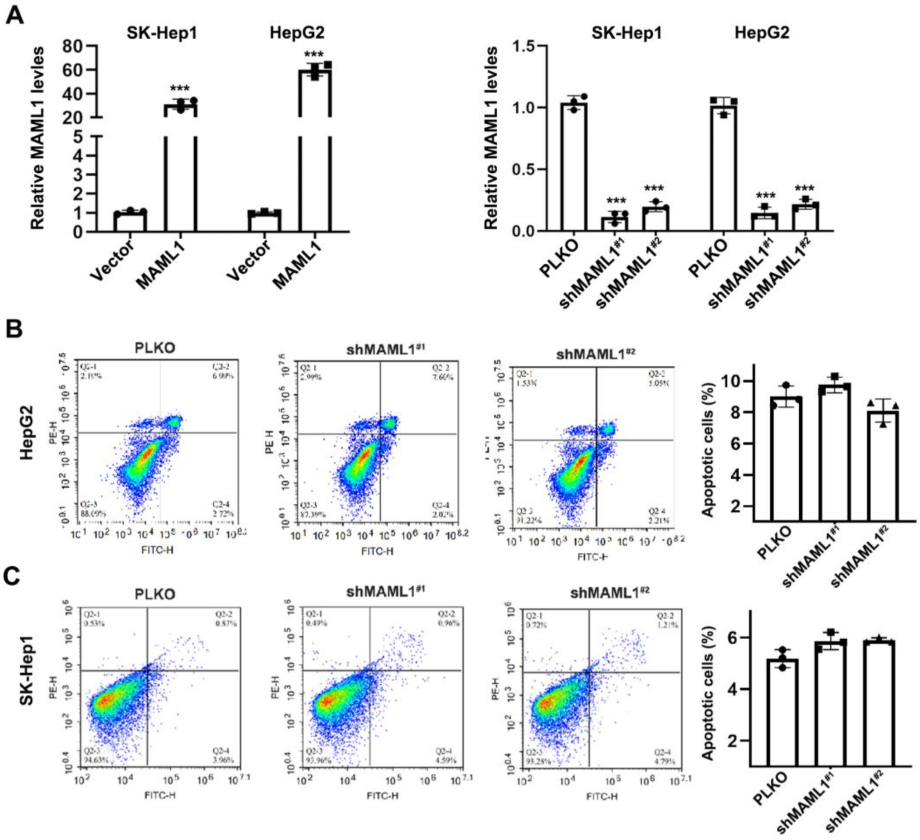

Supplementary Fig. 2 Validation of MAML1 manipulation efficacy and Its impact on apoptosis in HCC cells. **A.** RT–qPCR assay to detect the efficiency of MAML1 overexpression (Left) and MAML1 knockdown (Right) in both SK-Hep1 and HepG2 cells. **B-C.** MAML1 reduction had no effect on the apoptosis of HepG2 (B) or SK-Hep1 (C) cells. Left, representative images of the flow cytometry data. Right, the statistical analyses. The significance levels are denoted as ***p < 0.001.

Supplementary Figure 3

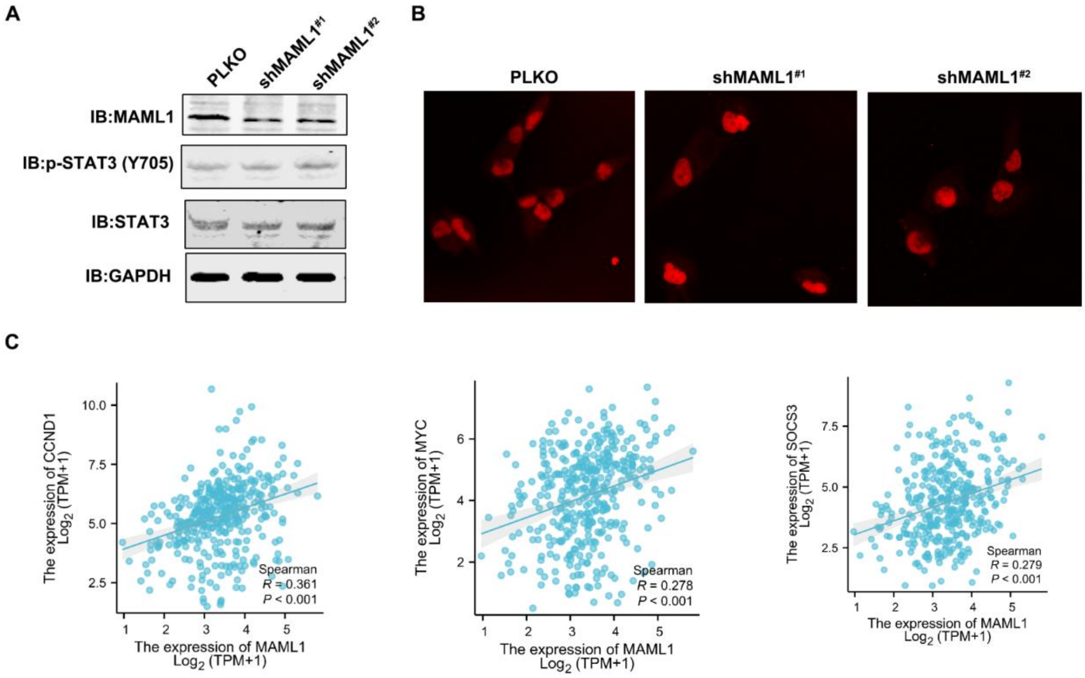

Supplementary Fig. 3 The impact of MAML1 on STAT3 nuclear translocation and the correlations between MAML1 and STAT3 downstream genes in TCGA-LIHC. **A.** Western blot analysis revealed that MAML1 knockdown did not affect the protein level or phosphorylation status (Y705) of STAT3 in HepG2 cells. GAPDH was used as a loading control. **B.** Immunofluorescence staining revealed that the nuclear translocation of STAT3 was not affected by MAML1. **C.** MAML1 expression was positively correlated with SOCS3, CCND1 and c-Myc expression in the TCGA-LIHC dataset.

Supplementary Figure 4

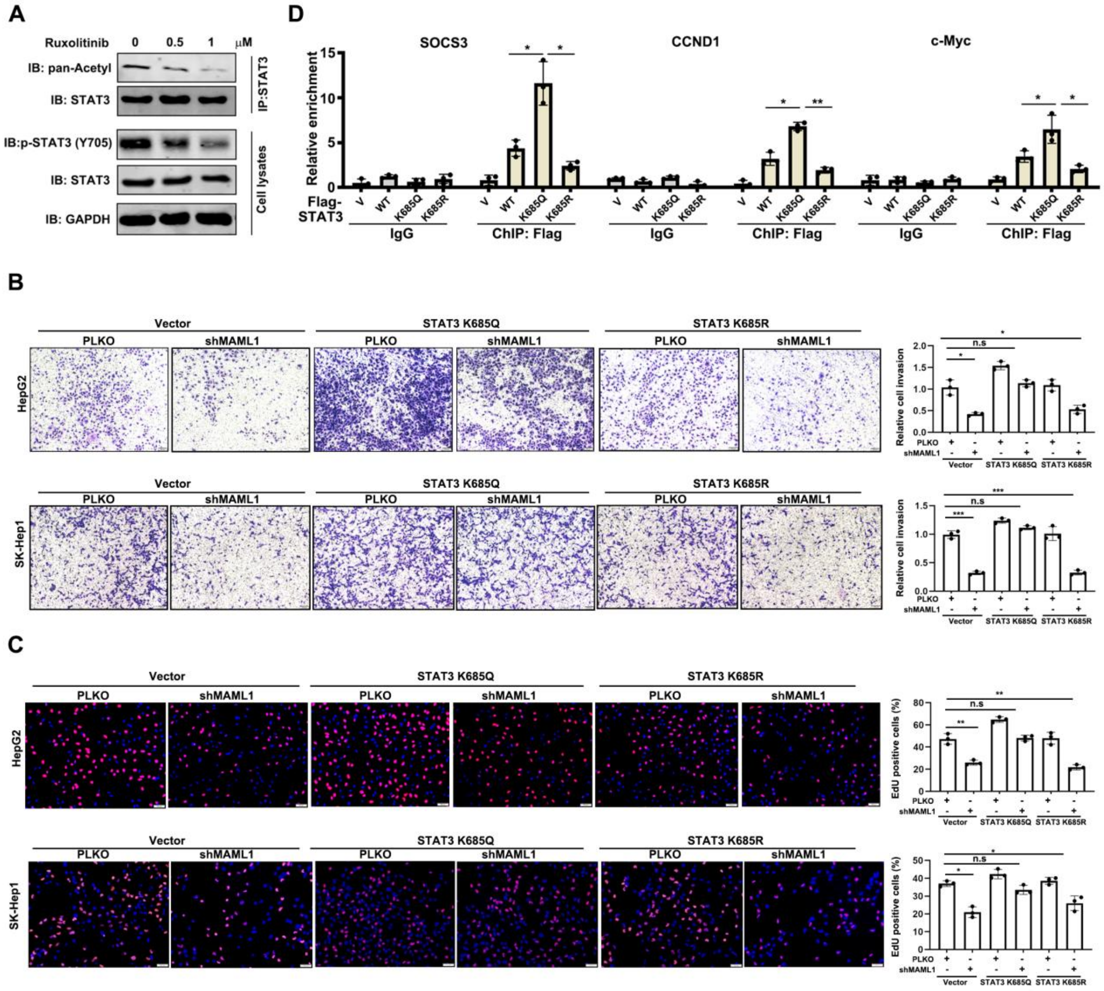

Supplementary Fig. 4 The oncogenic role of MAML1 in HCC acts through STAT3 acetylation. A. Western blot analysis revealed that ruxolitinib treatment could reduce the acetylation and phosphorylation levels of STAT3. GAPDH was a loading control. **B.** Chamber-Transwell invasion assays revealed that STAT3 K685Q but not K685R recovered shMAML1-reduced cell invasion of HepG2 and SK-Hep1 cells. Left, representative invading HCC cells. Right, the statistical analyses of the invading cells. **C.** EdU assays revealed that STAT3 K685Q but not K685R recovered shMAML1-reduced cell proliferation of HepG2 and SK-Hep1 cells. Left, representative EdU positive HCC cells. Right, the statistical analyses of the EdU positive cells. **D.** ChIP assays revealed the DNA binding abilities of STAT3 K685Q and K685R. *p < 0.05, **p < 0.01, and ***p < 0.001, and "n.s." indicates no significance.

Supplementary Figure 5

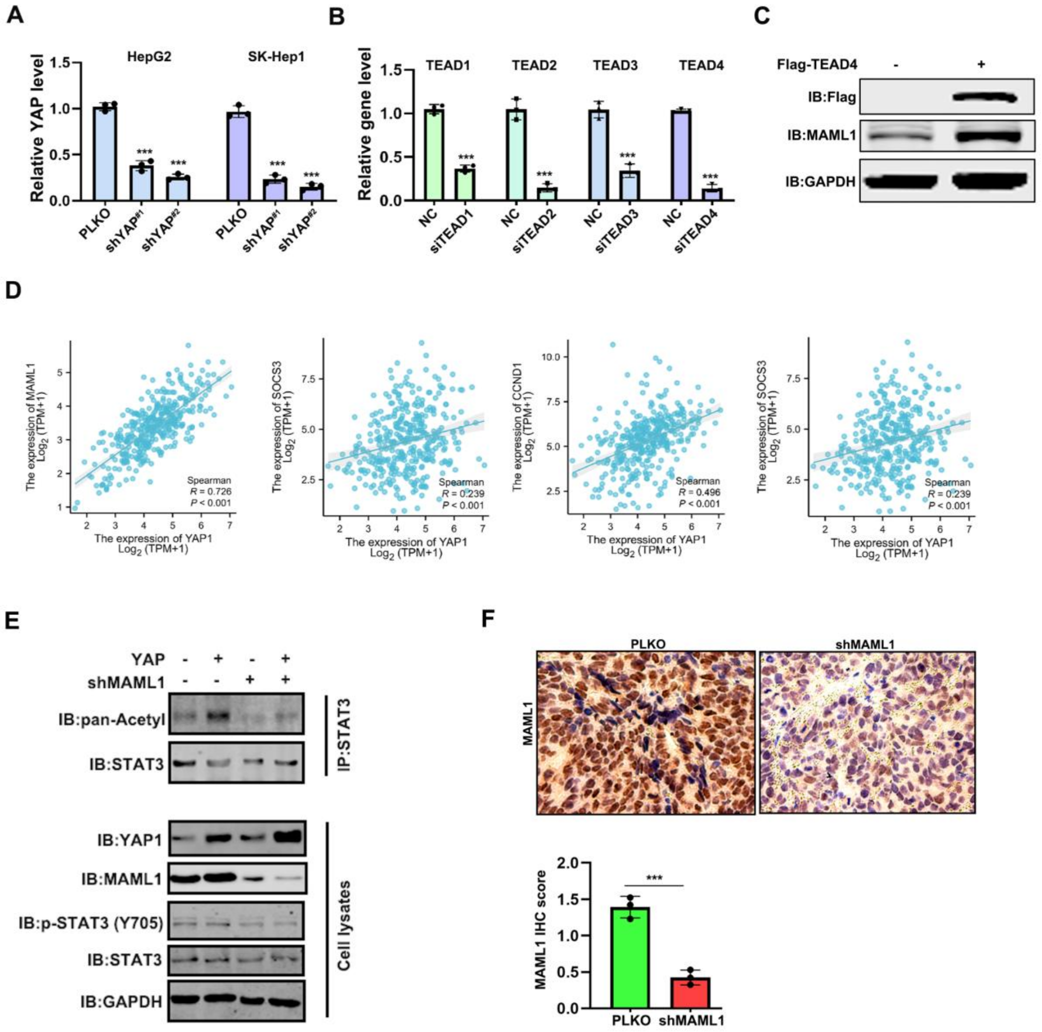

Supplementary Fig. 5 YAP-TEAD4 induced STAT3 activation via transcriptionally regulating MAML1 expression. A. Efficiency of YAP knockdown in HepG2 and SK-Hep1 cells. **B.** The knockdown efficiency of each TEAD member in HepG2 cells. **C.** Western blot analysis revealed that TEAD4 overexpression increased MAML1 expression in HepG2 cells. GAPDH was used as a loading control. **D.** YAP was strongly correlated with MAML1, SOCS3, CCND1 and c-Myc in the TCGA-LIHC cohort. **E**. Western blot analysis revealed the influence of YAP overexpression on the STAT3 acetylation levels w/o shMAML1 in HepG2 cells. **F.** IHC staining confirmed the knockdown efficiency of shMAML1 in vivo. Top, representative images. Bottom, quantification of the IHC staining. Significance levels are denoted as ***p < 0.001.

Supplementary Table 1. Sequences of primers, shRNAs or siRNAs

Primer Name	Sequence (5'-3')
MAML1-forward	GACTCTCTCAACAAAAAGCGTCT
MAML1-reverse	AGGAAATGACTCACTGGGGTTA
SOCS3-forward	CCTGCGCCTCAAGACCTTC
SOCS3-reverse	GTCACTGCGCTCCAGTAGAA
CCND1-forward	GCTGCGAAGTGGAAACCATC
CCND1-reverse	CCTCCTTCTGCACACATTTGAA
GAPDH-forward	GGAGCGAGATCCCTCCAAAAT
GAPDH-reverse	GGCTGTTGTCATACTTCTCATGG
MYC-forward	GGCTCCTGGCAAAAGGTCA
MYC-reverse	CTGCGTAGTTGTGCTGATGT
SOCS3 promoter-F	GCGCTCAGCCTTTCTCTG
SOCS3 promoter-R	GGAGCAGGGAGTCCAAGTC
CCND1 promoter-F	TCAGTCCCAGGGCAAATTCT
CCND1 promoter-R	CGGGAGAAACACACCTCTGA
MYC promoter F	TGAGTATAAAAGCCGGTTTTCG
MYC promoter R	CTGCCTCTCGCTGGAATTACTA
P1-forward	GTCTCGATCTCCTGACCTCG
P1-reverse	AAGGTGGAAGGATCGCTTGA
P2-forward	CCCTCCCATATTCTCCCGTC
P2-reverse	CTCTCTGCCAGTCTGTACCC
P3-forward	GATTCTTTGGCCGTCTGTCC
P3-reverse	CTCTGCAGTAGGGGACAACT
P4-forward	GTAATCGCGGTACTTTGGCA
P4-reverse	GGAAATGCAAAGGACGACGT
shMAML1#1	GACATGAAGGACCTGTTTAAT
shMAML1#2	CCCACACTGTCGGGCTTATAA
shSTAT3	CTCAGAGGATCCCGGAAATTT
shYAP1#1	GGCTGCCACCAAGCTAGATAA
shYAP1#2	TGAGATGGATACAGGTGATAC
siTEAD1 sense	GAUCAGACUGCAAAGGAUATT
siTEAD1 anti-sense	UAUCCUUUGCAGUCUGAUCTT
siTEAD2 sense	GUCUGAUGAAGGCAAGAUGTT
siTEAD2 anti-sense	CAUCUUGCCUUCAUCAGACTT
siTEAD3 sense	GCAAACAGGUGGUAGAGAATT
siTEAD3 anti-sense	UUCUCUACCACCUGUUUGCTT
siTEAD4 sense	GCAAGCAGGUGGUGGAGAATT
siTEAD4 anti-sense	UUCUCCACCACCUGCUUGCTT
